# Supplementary material for: Evolutionary features of microscopic damage in shale under unloading action
Source: PLoS One. 2025 Sep 10;20(9):e0329437. doi: 10.1371/journal.pone.0329437 (PMC12422503; doi:10.1371/journal.pone.0329437)
Supplement: S1 File — (DOCX) [file pone.0329437.s001.docx]

**
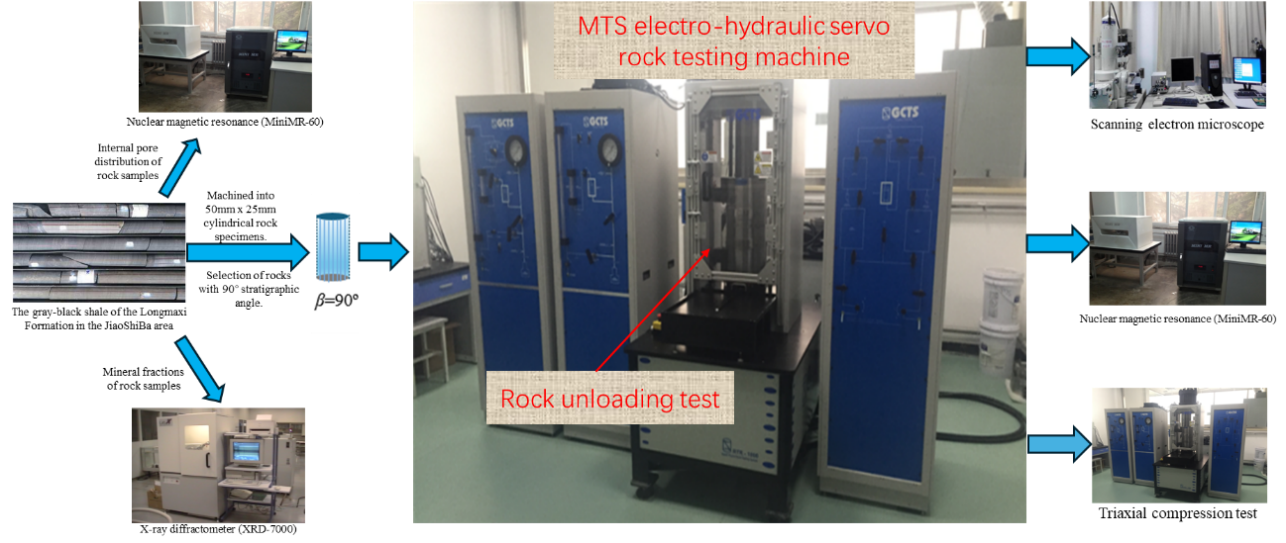
**

**Fig. 1** Experimental procedure flow.


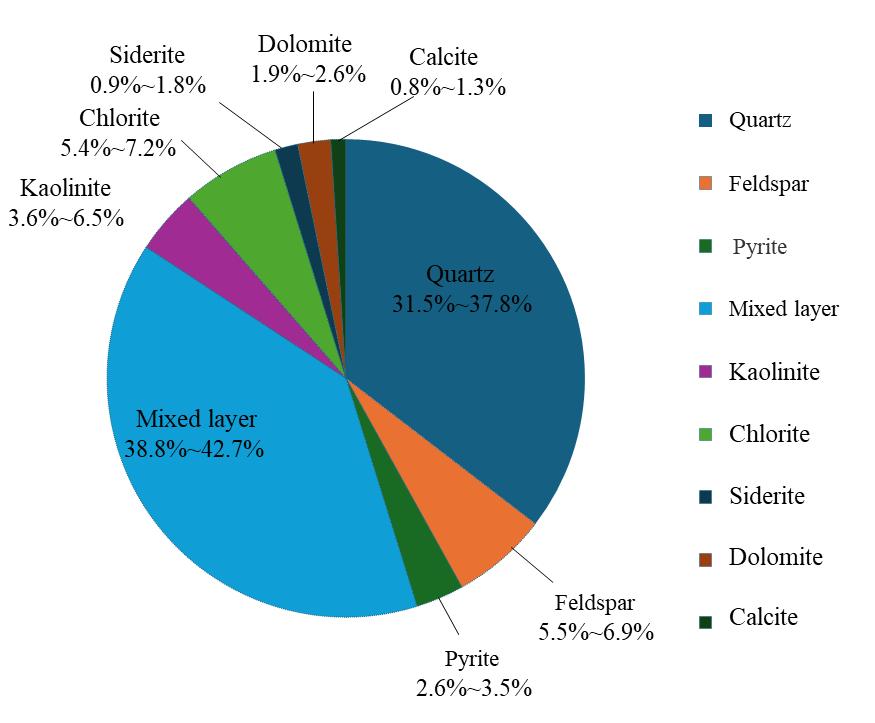


**Fig. 2** Mineral fractions of rock samples


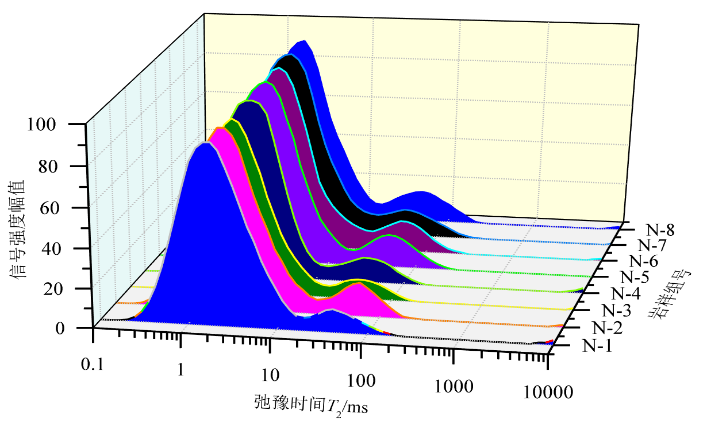


*T*_2_ relaxation time/ms

rock sample group number

Signal Strength Amplitude/ms^-1^

**Fig.** **3** *T*_2_ spectra distribution of samples


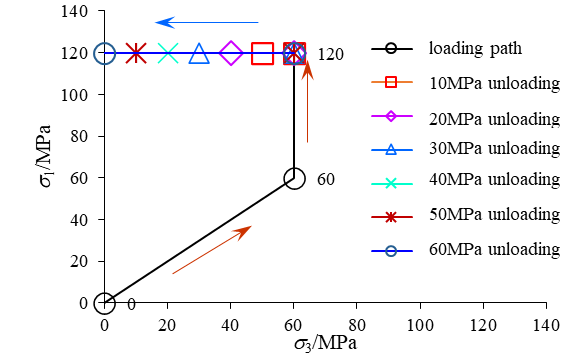


**Fig. 4** Schematic diagram of test stress unloading path


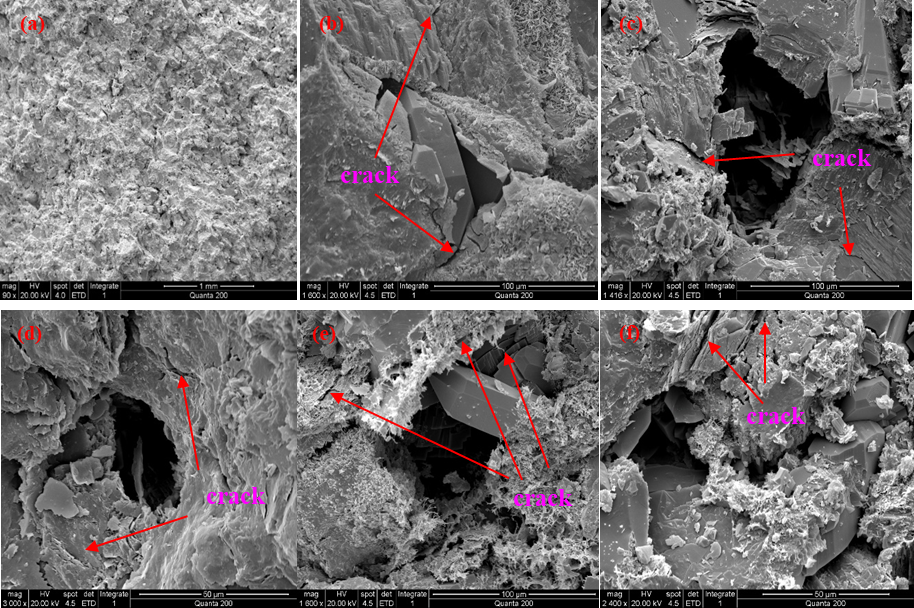


Note: (a) The unloading amplitude is 10MPa; (b) The unloading amplitude is 20MPa; (c) The unloading amplitude is 30MPa; (d) The unloading amplitude is 40MPa; (e) The unloading amplitude is 50MPa; (f) The unloading amplitude is 60MPa.

**Fig. 5** Images of SEM after unloading disturbance

**(f)**

**(e)**

**(d)**

**(c)**

**(b)**

**(a)**


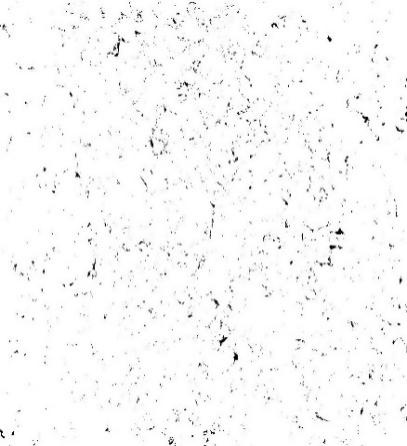

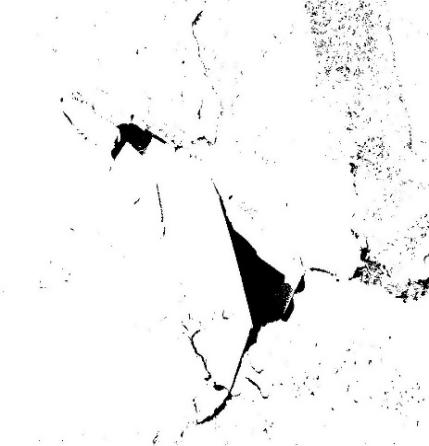

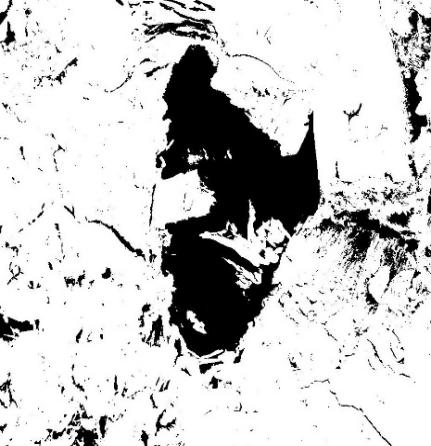

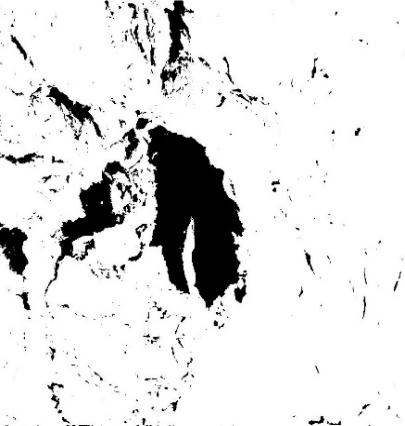

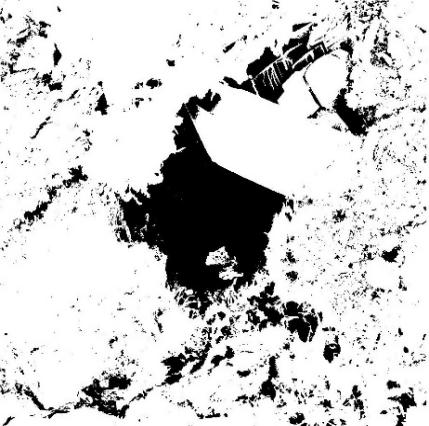

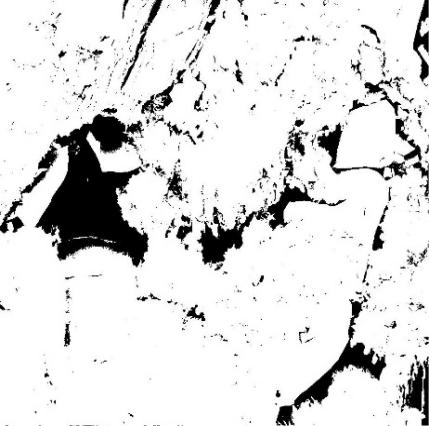


**Fig. 6** Images of SEM after binary processing

**(b)**

**(a)**


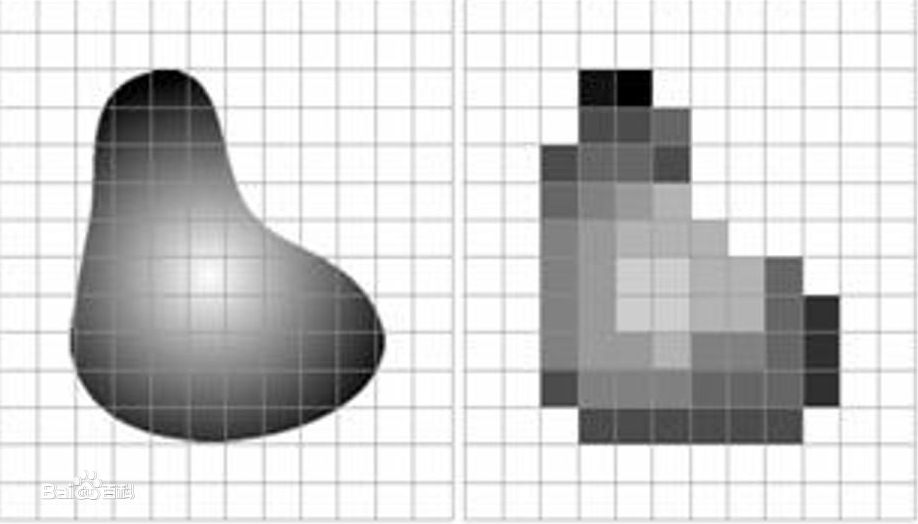

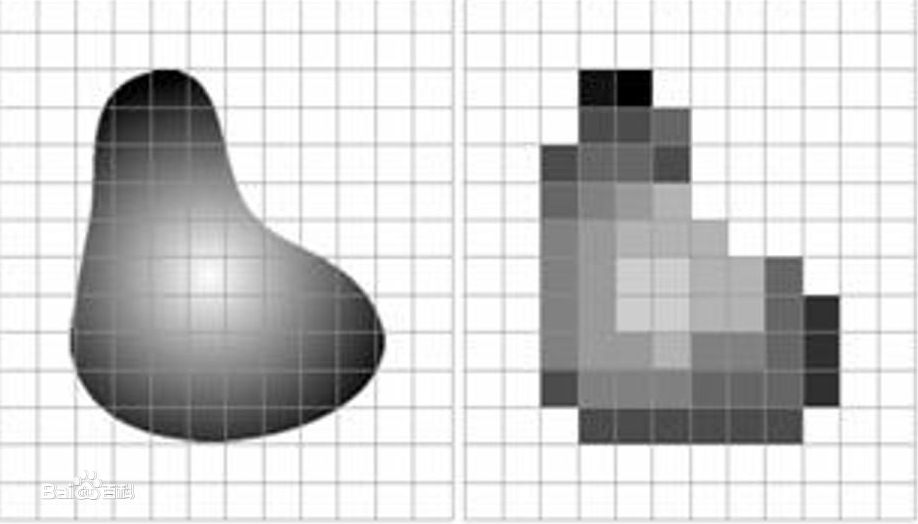


*M*

*N*

*O*

*M*

*N*

*O*

Note: (a) Continuous image that has been projected onto the sensor array; (b) Results of image sampling and quantization.

**Fig. 7** Image digitization process


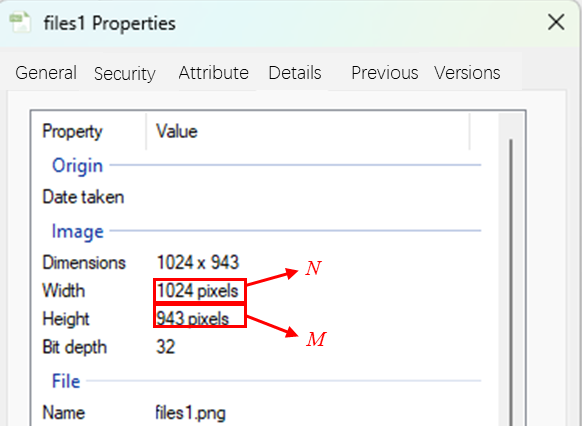


**Fig. 8** Image details


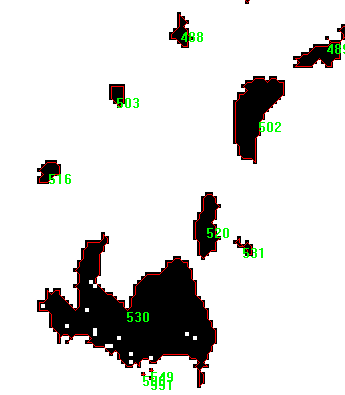


*S_i_*

*d*_max_

_Pore number_


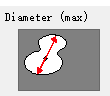

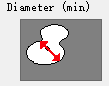

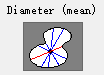


Maximum aperture

Minimum aperture

Average aperture size


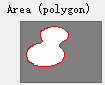


Pore area

**Fig.9** Statistics of pore structure parameters in selected areas  **Fig. 10** Aperture parameters


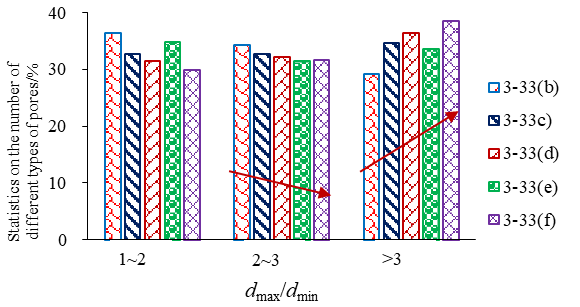


12(b)

12(c)

12(d)

12(e)

12(f)

**Fig.11** Number percentage of pores with different *d*_max_/*d*_min_ ratios


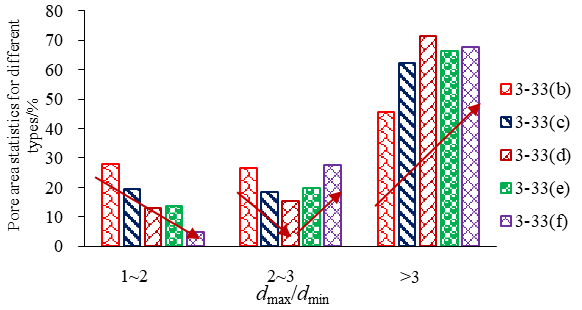


12(b)

12(c)

12(d)

12(e)

12(f)

**Fig.12** Area percentage of pores with different *d*_max_/*d*_min_ ratios


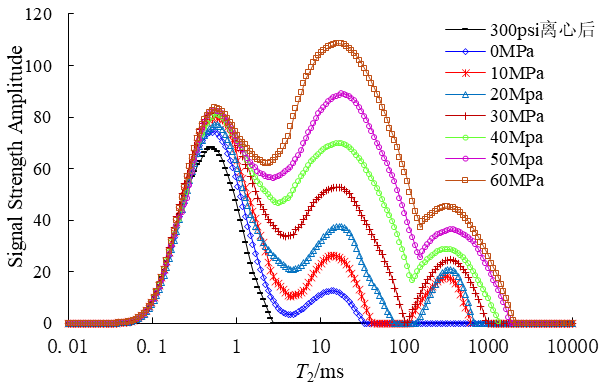


**Fig. 13** Shale *T*_2_ energy spectrum with different unloading amplitudes

**Fig. 14** *T*_2_ energy spectrum area growth rate after pores after different unloading amplitudes


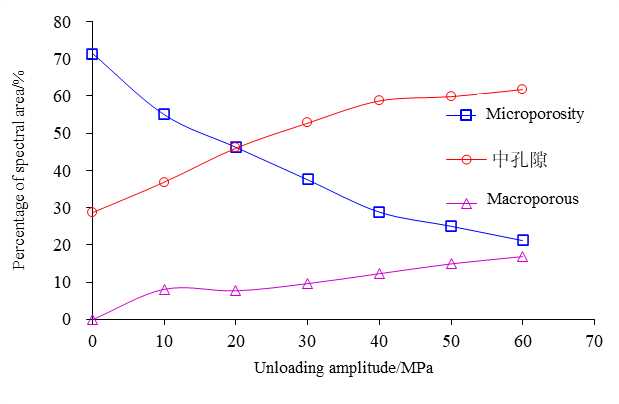


Mesopore

**Fig. 15** Proportion of various unloading amplitudes


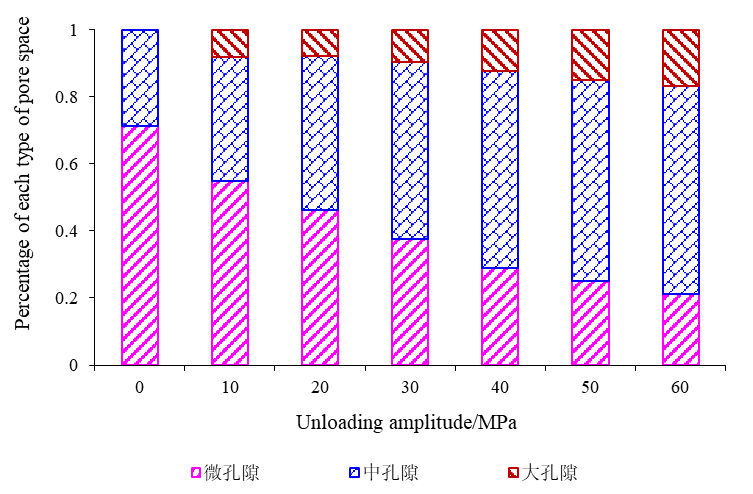


Mesopore

Macroporous

Microporosity

**Fig. 16** Percent stacking chart of micropore, mesopore, and macropore spectrum area


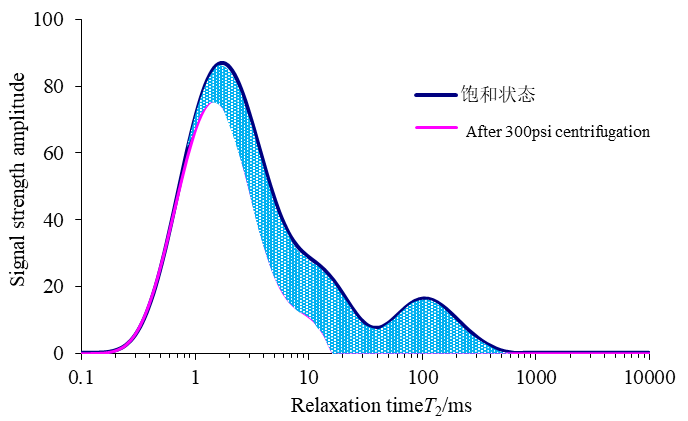


Saturation

**Fig. 17** *T*_2_ spectrum distribution in the saturated state and after centrifugation


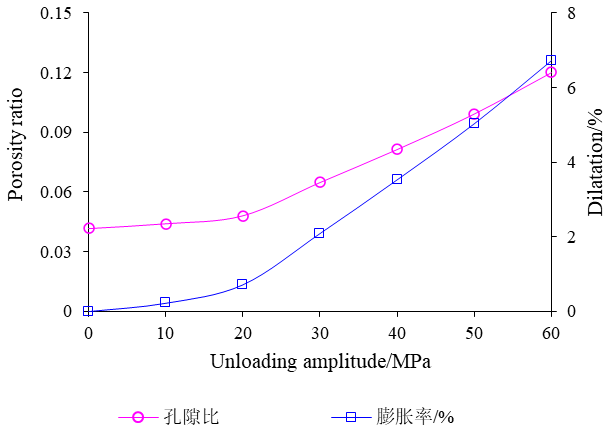


Dilatation/%

Porosity ratio

**Fig. 18** Variation of shale void ratio and expansion rate after unloading disturbance


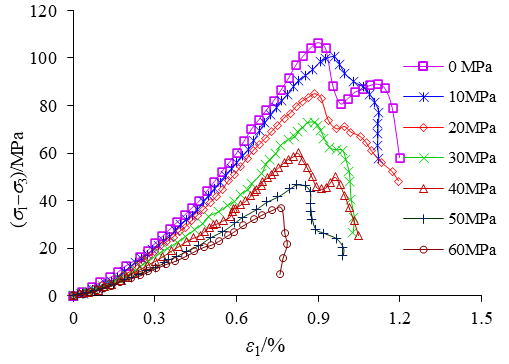


*σ*_1_

*σ*_3_=60MPa

*β*=90º

**Fig.19** Shale triaxial compression test after unloading disturbance


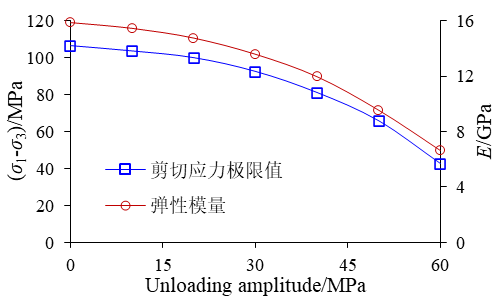


Shear stress limit value

Modulus of elasticity

**Fig. 20** Variation of shear strength and elastic modulus with different unloading amplitudes


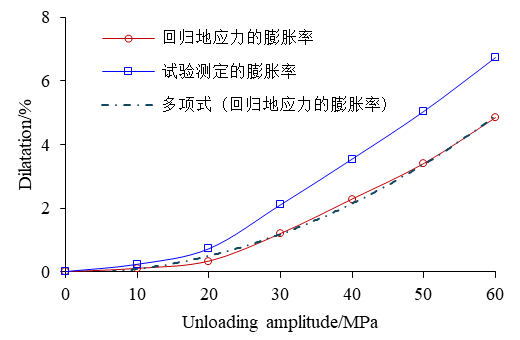


*η*_u_= 0.0014x^2^-0.0002x -0.0468
R^2^ = 0.9975

Expansion rate of regression ground stress

Polynomial

Test-measured expansion rate

**Fig. 21** Changing law of rock expansion rate with different unloading amplitude


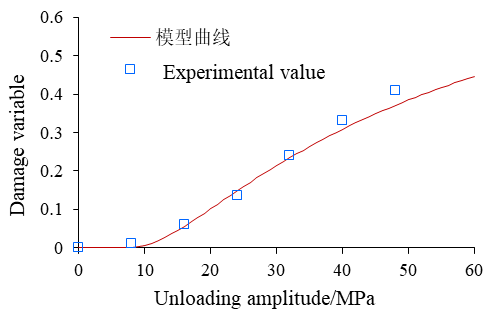


Model curve

**Fig. 22** Variation law of damage variables after unloading disturbance
